# Development and Validation of Miglitol and Its Impurities by RP-HPLC and Characterization Using Mass Spectrometry Techniques

**DOI:** 10.3390/scipharm84040654

**Published:** 2016-10-14

**Authors:** Kesavan Balakumaran, Mosesbabu Janagili, Nagaraju Rajana, Sureshbabu Papureddy, Jayashree Anireddy

**Affiliations:** 1 Analytical Research, Custom Pharmaceutical Services, Dr. Reddy’s Laboratories Ltd., Bollaram Road, Miyapur, Hyderabad 500049, India; mosesbabuj@drreddys.com (M.J.); nagarajurajana@drreddys.com (N.R.); psbabu8277@gmail.com (S.P.); 2 Centre for Chemical Sciences & Technology, Institute of Science and Technology, Jawaharlal Nehru Technological University, Kukatpally, Hyderabad 500085, India; jayashreeanireddy@gmail.com

**Keywords:** reverse phase liquid chromatography, miglitol, stability-indicating methods, forced degradation, high performance liquid chromatography, DNJ (1-deoxynojirimycin), mass spectrometry

## Abstract

Alpha glucoside inhibitors used to treat type-2 diabetes mellitus (DM) are likely to be safe and effective. These agents are most effective for postprandial hyperglycemia. Miglitol is a type of drug used to treat type-2 DM. A simple, selective, linear, precise and accurate reversed-phase high-performance liquid chromatography (RP-HPLC) method was developed and validated for a related substance of miglitol and its identification, and characterization was done by different mass spectrometry techniques. The gradient method at a flow rate of 1.0 mL/min was employed on a prevail carbohydrate ES column (250 × 4.6 mm, 5 μm particle size) at a temperature of 35 °C. Mobile phase A consisted of 10 mM dipotassium hydrogen orthophosphate adjusted to pH 8.0 using concentrated phosphoric acid and mobile phase B consisted of acetonitrile. The ultraviolet detection wavelength was 210 nm and 20 μL of the sample were injected. The retention time for miglitol was about 24.0 min. Forced degradation of the miglitol sample was conducted in accordance with the International Conference on Harmonisation (ICH) guidelines. Acidic, basic, neutral, and oxidative hydrolysis, thermal stress, and photolytic degradation were used to assess the stability-indicating the power of the method. Substantial degradation was observed during oxidative hydrolysis. No degradation was observed under the other stress conditions. The method was optimized using samples generated by forced degradation and sample solutions spiked with impurities and epimers. Good resolution of the analyte peak from peaks, corresponding to process-related impurities, epimers and degradation products, was achieved and the method was validated as per the ICH guidelines. The method can successfully be applied for routine analysis of miglitol.

## 1. Introduction

Diabetes mellitus (DM) is simply known as diabetes, a lifelong, progressive disease, which is a chronic metabolic disorder with steadily increasing prevalence all over the world. The relative deficiency of insulin secretion and varying degrees of insulin resistance is characterized by high circulating glucose levels [1]. As a result of this trend, it is quickly becoming an epidemic in poorly developed countries, with the number of people affected, expected to double in the next decade, due to an increase in the aging population, thereby adding burden to the existing health care providers [2]. No cure has been found yet for the disease; however, treatment modalities including lifestyle modification, treatment of obesity, oral hypoglycemic agents and insulin sensitizers that reduce insulin resistance are still recommended as first line treatment [3]. Recent research in pathophysiology of type 2 DM led to the introduction of new medications like glucagon, peptide 1 analogues, dipeptidyl peptidase-IV inhibitors, glucagon-receptor antagonists and metabolic inhibitors of hepatic glucose output.

Miglitol ((2*R*,3*R*,4*R*,5*S*)-1-(2-hydroxyethyl)-2-(hydroxymethyl)piperidine-3,4,5-triol) is an oral anti-diabetic drug and an inhibitor of the intestinal α-glucosidase. The inhibition of the glucosidase activity in the intestinal brush border blocks the breakdown of starch and disaccharides to absorbable monosaccharides, which results in a decrease in the intestinal absorption of starch, disaccharides and dextrin, leading to carbohydrate malabsorption and blunting of the postprandial rise in blood glucose. Miglitol was approved for use in the United States in 1999 and it was the second α-glucosidase inhibitor (after acarbose) introduced into clinical practice. The current indications include the management of glycemic control in type 2 diabetes, used in combination with diet and exercise, with or without other oral hypoglycemic agents or insulin. Miglitol is available generically and under the brand name Glyset (Pfizer, New York, NY, USA) in tablets of 25, 50 and 100 mg. The typical initial dose in adults is 100 mg with each meal (with the first bite) followed by a gradual increase. Miglitol causes malabsorption and gastrointestinal side effects are common including flatulence, diarrhea and abdominal boating.

Few analytical methods have been reported for the estimation of miglitol in bulk drug substance, tablets and the quantification of miglitol in biological samples. In order to follow the current good manufacturing practice of pharmaceuticals products, during the chemical synthesis of active ingredients, the possible impurities of reagents or starting materials have to be quantified to the lower limit based on the dosage of active ingredients [4,5,6]. The reported analytical method employs the quantification by ultra-performance liquid chromatography – electrospray ionisation mass spectrometry (UPLC-ESI-MS), high-performance liquid chromatography – ultra violet detector (HPLC-UV), capillary electrophoresis and high-performance liquid chromatography – evoprative light scattering detector (HPLC-ELSD) [5,6,7,8,9,10]. Some of the analytical methods reported for the determination of miglitol and its derivatives in the blood plasma have been previously described [11,12,13,14,15,16,17].

With the available information, no method has been reported for the determination of miglitol, 1-deoxynojirimycin (DNJ), its epimers, its potential impurities and its degradants in the drug substance using HPLC. The objective of this research work is to develop a simple stability-indicating liquid chromatographic method for the related substance. The forced degradation was performed as per International Conference on Harmonisation (ICH) recommended conditions, i.e., acid, base and water hydrolysis, oxidative, thermal and photolytic stressed conditions to prove the stability-indicating ability of the method. The mixture of the degraded sample and its related impurities were used to optimize the method. The method was also validated as per current regulatory bodies’ requirements [18,19,20,21,22,23]. Mass spectrometry analysis was also performed using the liquid chromatography mass spectrometry (LCMS) compatible HPLC condition to determine the molecular weight of the impurities eluted by HPLC.

## 2. Materials and Methods

### 2.1. Chemicals and Reagents

Miglitol and its impurities were synthesized and purified using column liquid chromatography by the process research department of Custom Pharmaceutical Services of Dr. Reddy’s laboratories limited, Hyderabad, India. Acetonitrile (HPLC-grade) and analytical reagent grade dipotassium hydrogen phosphate, sodium hydroxide, hydrochloric acid and hydrogen peroxide were purchased from Rankem (Mumbai, India) and used for the studies. Millipore Milli Q plus (Bangalore, India) purification system was used to prepare high pure water.

### 2.2. Equipment

The method development attempts, forced degradation studies and the method validation were performed using Agilent 1100 series LC system with a diode array detector (Agilent Technologies, Santa Clara, CA, USA). The data were collected and processed using chemstation software (Agilent Technologies). The photolytic degradation was carried out using Binder KBS240 photolytic chamber (Bohemia, NY, United States), The peak homogeneity was established in Agilent LCMS 6410 QqQ instrument (Agilent Technologies).

#### 2.2.1. Mass Spectrometry

The mass spectra of miglitol, epimeric impurities and DNJ were recorded with a LC-MS 6410 QqQ instrument (Agilent Technologies). For LC-MS identification of the impurities, the HPLC method given above was used by developing a LC-MS compatible method, applying 0.1% acetic acid as mobile phase A and acetonitrile as mobile phase B with gradients of 0/90, 35/70, 40/70, 50/90, 55/90 (time/%B). The samples of the synthesized compounds were directly injected, using a syringe, at a concentration of (acetonitrile:water, 50:50, %*v*/*v*). High resolution mass spectral data were recorded using Waters UPLC-TOF with LCT Premier XE Mass Lynx TM software (Milford, MA, USA). The conditions used were as follows: capillary voltage at 2300 V, sample cone voltage 80 V, dissolution temperature 250 °C, source temperature 120 °C, desolvation gas flow 500 L/h and cone gas flow 50 L/h.

#### 2.2.2. Chromatographic Conditions

The chromatographic separation was optimized in the prevail carbohydrate ES column (W. R. Grace & Co.-Conn., Columbia, MD, USA) with the dimension of 250 mm × 4.6 mm and 3.5 µm particle size. The elution involved 10 mM dipotassium hydrogen orthophosphate in water and pH adjusted to 8.0 using concentrated phosphoric acid buffer as mobile phase A and acetonitrile as mobile phase B with gradient of 0/90, 35/70, 40/70, 50/90, 55/90 (Time/%B). The flow rate of the mobile phase and the column temperature was set as 1.0 mL·min^−1^ and 35 °C. The detection wave length was optimized at 210 nm. The column loading was finalized by injecting 20 µL of miglitol (10 mg/mL) to the HPLC. A mixture of water and acetonitrile was used as a diluent in the same ratio.

#### 2.2.3. Preparation of Solutions

Miglitol solution was prepared at a target analyte concentration (TAC) of 10,000 µg·mL^−1^ in the diluent for related substances determination and assay determination. A stock solution with the blend of impurity A, B, C, D, E and F was also prepared in the same diluent for the preparation of the system suitability solution which contained 0.15% (*w*/*w*) of each impurity with respect to TAC of miglitol.

#### 2.2.4. Standard and Sample Preparation

##### Preparation of standard stock solution-1

Weighed 50 mg of miglitol and its impurities A, B, C, D, E and F in a 25 mL volumetric flask containing 15 mL of diluent (acetonitrile:water, 50:50, %*v*/*v*), and made up to the mark with diluent.

##### Preparation of standard stock solution-2

Transferred 1.2 mL of standard stock solution-1 into a 25 mL volumetric flask containing 15 mL of diluent and made up to the mark with diluent.

##### Preparation of 0.15% (*w*/*w*) standard solution

Transferred 1.6 mL of standard stock solution-2 into a 10 mL volumetric flask containing 3 mL of diluent and made up to the mark with diluent (with resepect to 10 mg/mL).

##### Preparation of 0.05% (*w*/*w*) standard solution (Limit of quantitation (LOQ) solution)

Transferred 3.3 mL of 0.15% (*w*/*w*) standard solution into a 10 mL volumetric flask containing 3 mL of diluent and made up to the mark with diluent (with respect to 10 mg/mL).

##### Preparation of 0.02% *w*/*w* standard solution (Limit of detection (LOD) solution)

Transferred 3.3 mL of 0.05% (*w*/*w*) standard solution into a 10 mL volumetric flask containing 3 mL of diluent and made up to the mark with diluent (with respect to 10 mg/mL).

##### Preparation of miglitol sample solution

Weighed 100 mg of miglitol into a 10 mL volumetric flask containing 3 mL of diluent (acetonitrile:water, 50:50, %*v/v*), and made up to the mark with diluent.

### 2.3. Method Development

The core objective of the chromatographic method is the detection and separation of all the known and degraded impurities of miglitol with a better baseline [16]. Deoxynijomycin is the intermediate precursor which is the potential impurity in the synthesis process of miglitol. Other epimeric impurities were possible in the process due to epimerization of DNJ. Other epimers of miglitol were synthesized by using the respective starting material. The final stage involved alkylation of DNJ using bromoethanol as alkylating agent. Due to the chromophore change, the λ max. changed from 205 nm to 210 nm during this alkylation. At 205 nm, there was low absorption signal response for the precursor and the related impurities. At 210 nm, all the intermediates and related impurities had a good response and also miglitol had a reasonable response. Crude samples of miglitol and all related impurities were quantified against miglitol and found that the mass balance was close to 100%, which is supporting the selection of 210 nm.

Initial attempts for the method development were made in water and acetonitrile as mobile phases on a C18 reverse phase column, miglitol, DNJ and epimers of miglitol were eluted early at void volume.

**Figure 1:**
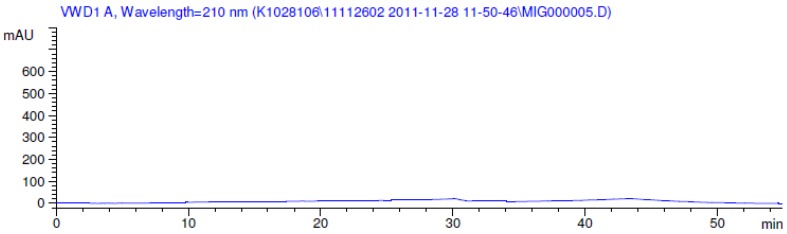
Blank chromatogram.

**Figure 2:**
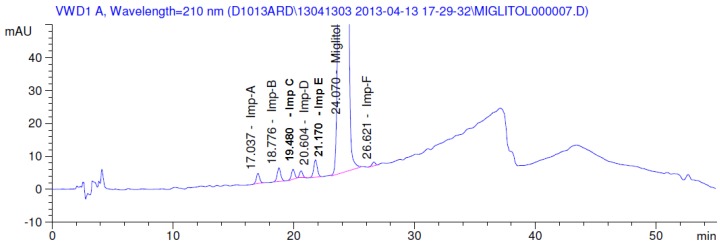
Typical system suitability test chromatogram.

Latter attempts for the method development were made using potassium hydrogen phosphate as mobile phase in an Acclaim mixed mode column (Thermo Fisher Scientific Inc., Waltham, MA, USA). Miglitol and DNJ were not separable from each other and with epimers is almost close retention. However, good peak shape and the resolution of all the related impurities was achieved using a prevail carbohydrate-ES column with the dimension of 250 mm × 4.6 mm and 3.5 µm particle size, by using solutions A and B as mobile phase. Though the selected stationary phase was not end-capped, there was no interaction between the analyte and the stationary phase. The selected stationary phase was very stable even at the highly basic pH, present when ammonia was used as a mobile phase. Solution A contained dipotassium hydrogen orthophosphate at pH 8.0 adjusted with orthophosphoric acid and solution B contained acetonitrile. The flow rate of the mobile phase was 1.0 mL·min^−1^. The gradient program was optimized to get the required retention of miglitol, DNJ and miglitol epimeric impurities. The HPLC gradient program was set as: time/% solution B: 0/90, 35/70, 40/70, 50/90, and 55/90 with a gradient delay of 5 min. The column temperature was set to 35 °C in order to reduce the back pressure of the column with the optimized gradient program and the peak shape of DNJ, miglitol, and its epimeric impurities was improved. In the optimized conditions, it was observed that miglitol and its epimers (Impurity B, D and E), DNJ (Impurity F), monoalkylated miglitol (Impurity C) and dialkylated miglitol (Impurity A) were well separated with a resolution greater than 1.5 and no interference of blank (Figure 1 and Figure 2). The system suitability resulted within the acceptance criteria (Table 1) and the developed LC method was found to be specific for miglitol, its known impurities and its degradation impurities. The structure of the miglitol active pharmaceutical ingredient and its impurities namely A, B, C, D, E, F and miglitol *N*-Oxide impurity are represented in Table 2.

#### 2.3.1. Method Validation

During method optimization, all chromatographic parameters were found to prove specificity, precision, linearity, accuracy, robustness and solution and mobile phase stability of active ingredients and its impurities.

#### 2.3.2. Specificity

Specificity is the ability of the method to measure the analyte in the presence of its potential impurities; they might be process related or degradation impurities. The specificity of the developed liquid chromatographic method for miglitol was established in the presence of its known impurities namely deoxynirjomycin (DNJ), miglitol empimers (imp-B, D, E and F), monalkyalted miglitol (imp-C) and di alkylated miglitol (imp-C) and miglitol degradation product. Forced degradation studies were performed on miglitol to grant the signal of the stability-indicating property and specificity of the developed method. The stress conditions engaged for degradation studies, as per the ICH recommended conditions, included photolytic, thermal, oxidation and hydrolysis in acid, base and water. The photolytic stressed studies were performed following ICH Q1B guidelines [19]. Samples were exposed for 11 days to 1.2 million lux hours for visible and 200 Wh/m^2^ for ultraviolet [19]. The thermal stress was done at 105 °C for 10 days. The acid, base stress was performed with 0.5 N HCl and 0.5 N NaOH at the concentrated sample solutions for 10 days at room temperature (25 ± 2 °C) and further dilution of the analyte concentration was performed for the quantification of miglitol and its degradants. Water hydrolysis was performed for 10 days at room temperature. The oxidation stress was done for 1 h at room temperature [19]. Peak purity of stressed samples of miglitol and the spiked solutions of Impurity A, Impurity B, Impurity C and epimers were checked by Agilent 1100, diode array detector (DAD) (Agilent Technologies).

#### 2.3.3. Precision

The precision of an analytical procedure expresses the closeness of agreement between a series of measurements from multiple sampling of the homogenous sample under prescribed conditions.

Six individual solution preparations were made with each of the known impurities at 0.15% (*w*/*w*) level. Quantification of individual impurities and miglitol was done for each of the preparations and the percentage of relative standard deviation RSD of the content of the impurities was determined. Method precision experimentation was repeated with different lots of column and different instrument in the same laboratory to evaluate the intermediate precision.

#### 2.3.4. Linearity

The linearity of an analytical test procedure is its ability to obtain test results within the given range, which is directly proportional to the concentration of the analyte in the sample. The linearity of the method was established for miglitol and its impurity. The solution of miglitol and its known impurities was prepared at five different concentrations from 0.05% to 0.30% (*w*/*w*) of analyte concentration. The regression line was plotted with area versus concentration using the method of least-squares analysis. The values of the slope and *Y*-intercept of the plot were calculated.

#### 2.3.5. Accuracy

Accuracy of impurities at each level was established by standard addition of the known quantities of impurities in test sample and calculating the recovery. The study was carried out in triplicate at LOQ, 0.075, 0.15 and 0.225% (*w*/*w*) of the TAC. The recovery of impurities was calculated by calculating the amount of impurities spiked and the amount of the impurity calculated from the spiked sample.

#### 2.3.6. Solution Stability and Mobile Phase Stability

The solution stability was measured by keeping both test solutions and impurities at 0.15% specification level in tightly capped volumetric flasks at room temperature for 48 h. The sample solutions were analysed at initial, 24 h, 48 h. The stability of the mobile phase was also measured for 48 h by analysing the freshly prepared reference solutions at initial, 24 h and 48 h. The mobile phase was kept constant during the study.

#### 2.3.7. Robustness

The robustness of an analytical procedure is a measure of its capacity to remain unaffected by small, but deliberate variations in method parameters and provides an indication of its reliability during normal usage. The flow rate of the mobile phase was 1.0 mL/min. To study the effect of flow rate on the system precision, it was changed by 0.1 units to 0.9 mL/min and 1.1 mL/min, while mobile phase components were held constant and the effect of flow rate was studied. The column temperature was studied by changing the 50 °C mentioned in the chromatographic conditions to 45 °C and 55 °C and it was found that the results did not change.

## 3. Results and Discussion

### 3.1. Mass Spectrometry Interpretation of Miglitol, 1-Dzeoxynojirimycin and Other Impurities

**Figure 3:**
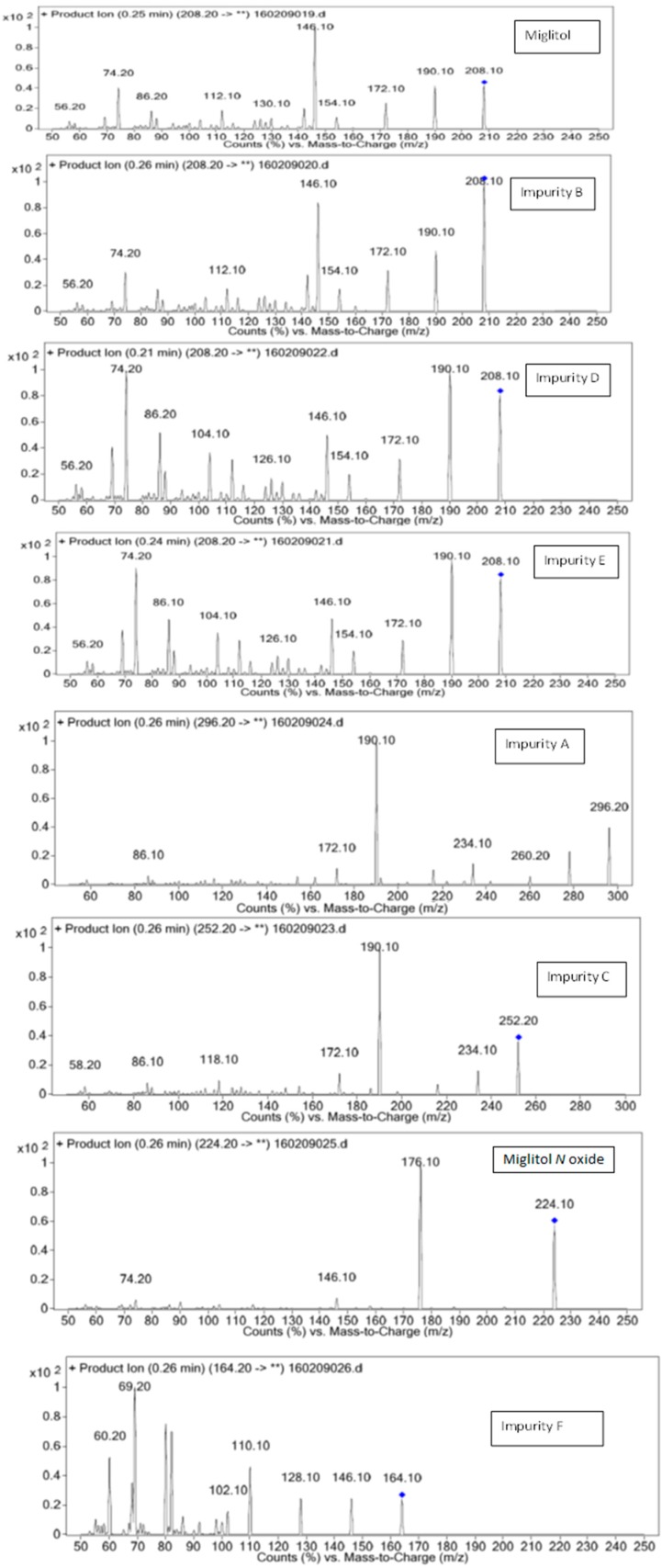
Fragmentation spectral data for miglitol and its impurities.

**Figure 4:**
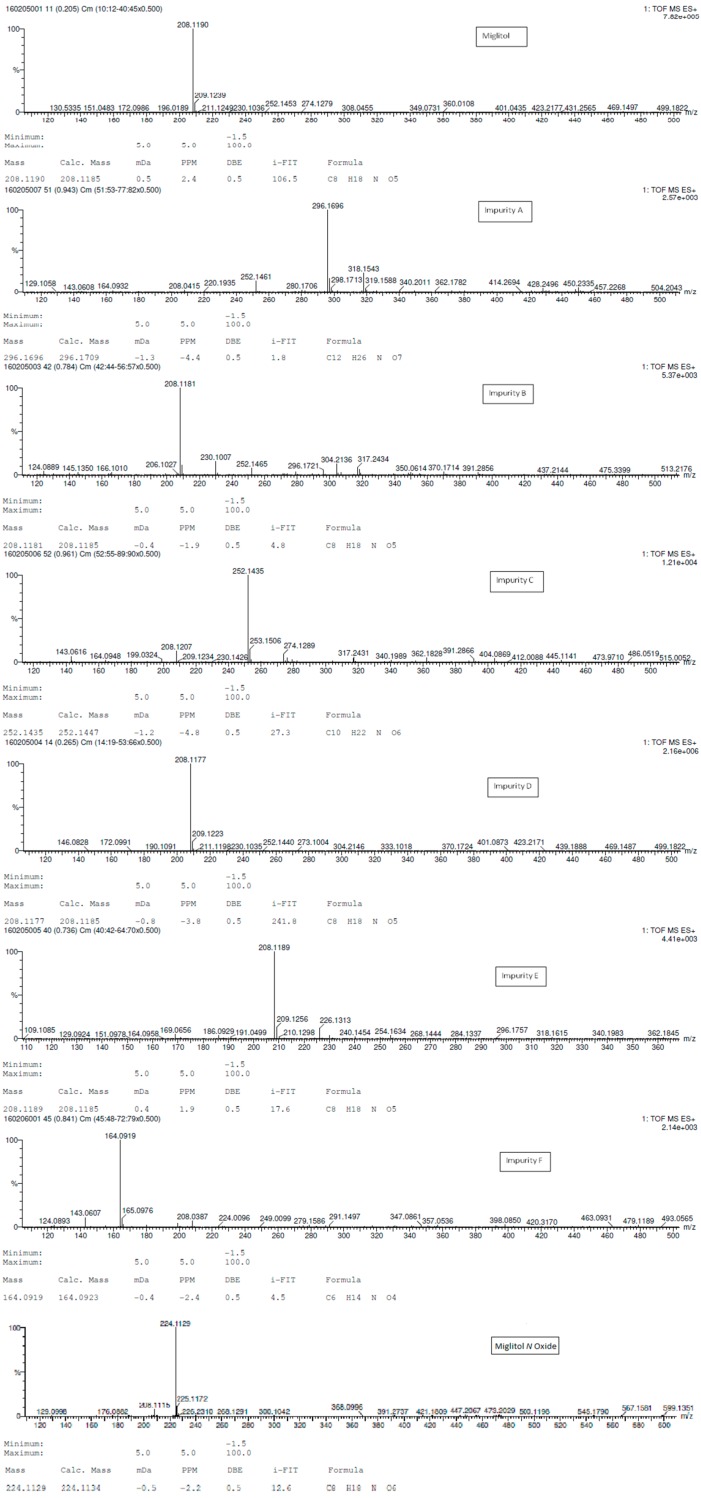
HRMS spectral data for miglitol and its impurities.

Structural characterization of miglitol and all the other impurities was performed using various mass spectral data by determining the molecular ion and its fragmentation. Electrospray ionization and triple quadrupole mass spectrometry was used to determine the m/z ratio of the molecular ions of all the structures using fragmentor voltage of 10 with a collision energy of 0 eV. The molecular ion [M + H]^+^ reported in Table 3 confirms the structure. In addition, fragmentation for each compound was studied using tandem mass spectrometry (MS/MS) by varying the collision energy and fragmentor voltage. High pure nitrogen gas was used as collision gas to get more accurate fragmentation data using electrospray ionization with fragmentor voltage at 135 and collision energy 20 eV (Figure 3). The MS/MS spectral data showed a similar finger print region for miglitol and its epimers. Due to the cyclic moiety, multiple fragments are observed in the spectral data. However, the retention time for all the compounds could be differentiated in the HPLC chromatogram showing the specificity of the LC method. However, for impurity A and impurity C the extension of aliphatic chain was confirmed by getting base peak at 190.1 and additional peak at 234.1 with the same fragmentation voltage and collision energy. The fragmentation was also studied with high collision energy 50 eV, in which many smaller fragmentation peaks were observed making the interpretation of the fragmentation pattern more complex. Hence, initial energy (20 eV) data is more appropriate rather than the later (50 eV). In the case of miglitol *N*-oxide impurity, when molecular ion 224.1 was subjected to MS/MS, a strong base signal of 176.1 was observed. This might be due to fragmentation of the aliphatic chain in C1. The high resolution mass spectral data (Figure 4) of miglitol and all the other compounds was studied. The exact mass of the compound is reported in Table 4. The fact that it shows less than 5 ppm difference, confirms that the structure and double bond equivalence (DBE) derived from the high resolution mass spectrometry HRMS instrument also matches the structure.

### 3.2. Results of Forced Degradation Studies

**Figure 5:**
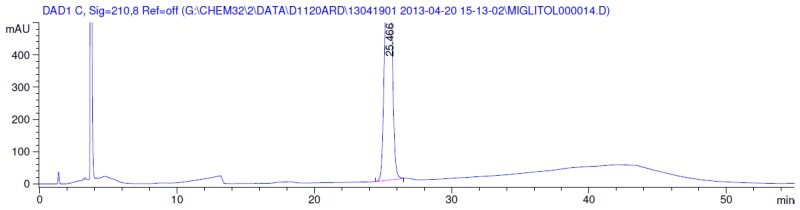
Chromatogram in base degradation.

**Figure 6:**
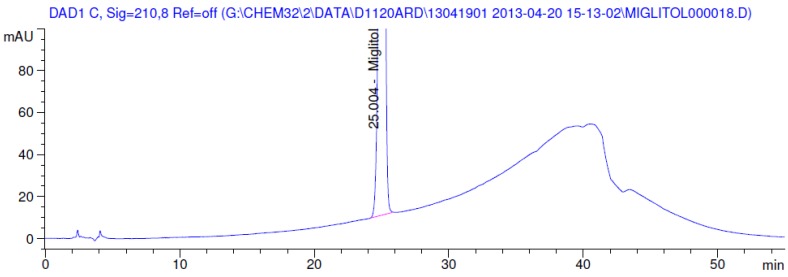
Chromatogram in thermal degradation.

**Figure 7:**
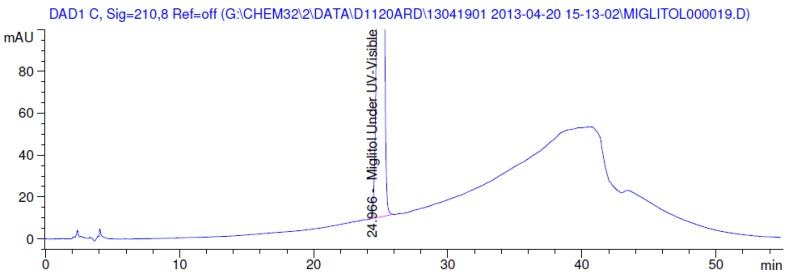
Chromatogram in ultraviolet (UV)-visible degradation.

**Figure 8:**
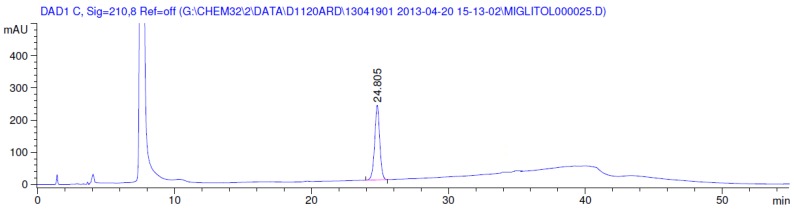
Chromatogram in oxidative degradation.

**Figure 9:**
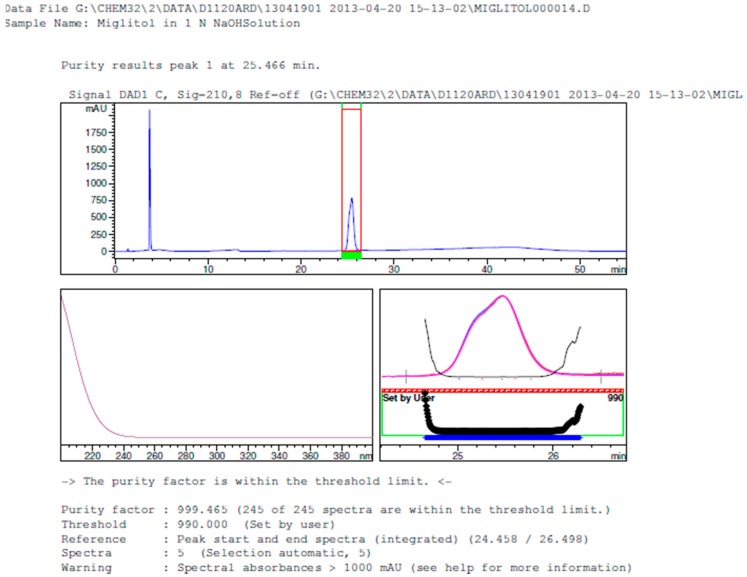
Peak purity in base degradation by diode array detector.

**Figure 10:**
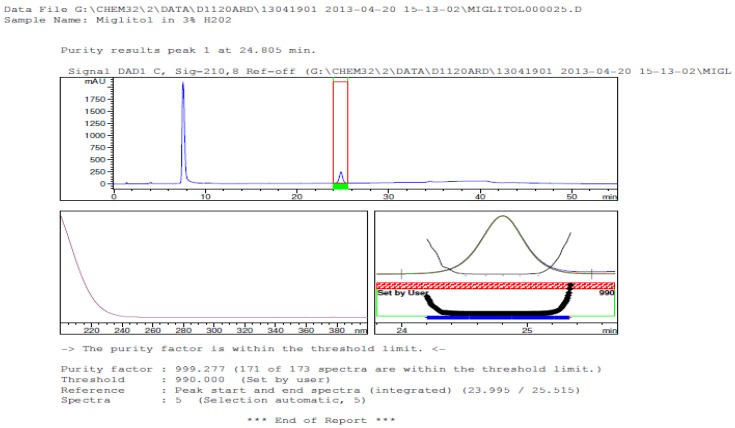
Peak purity in oxidative degradation by diode array detector.

**Figure 11:**
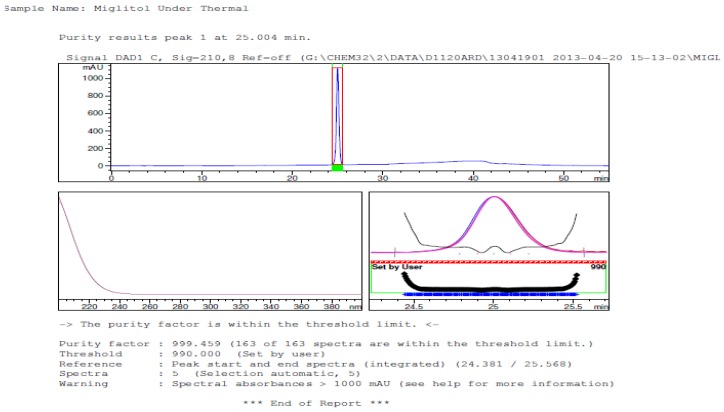
Peak purity in thermal degradation by diode array detector.

**Figure 12:**
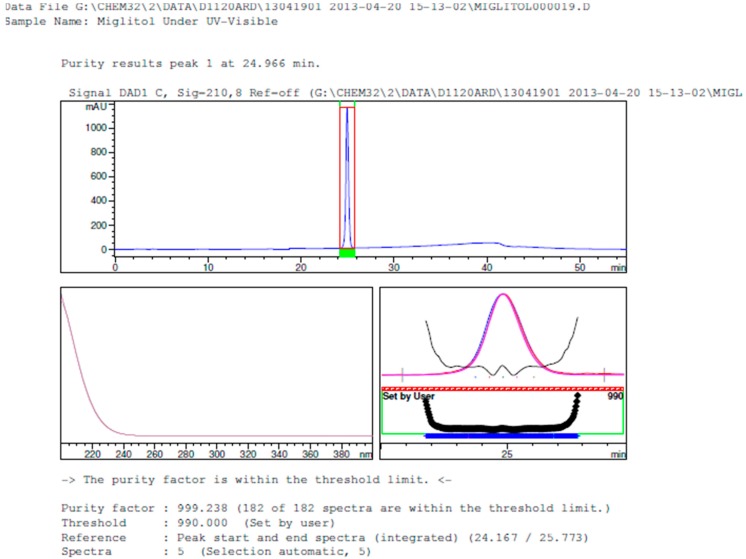
Peak purity in UV-visible degradation by diode array detector.

Degradation was not observed in stressed conditions when the analyte was subjected to photolytic, thermal, acid, base or water hydrolysis. The degradation of the drug was observed only under oxidative conditions. Miglitol under oxidative conditions leaded to the formation of one unknown degradation peak at the relative retention time (RRT) of 0.33. The peak purity factor, over the threshold obtained in all stressed samples for the analyte peak, demonstrated its specificity (Figure 5, Figure 6, Figure 7, Figure 8, Figure 9, Figure 10, Figure 11 and Figure 12). The summary of the forced degradation is captured in Table 5. The mass of the degradant is 16 units higher than the molecular weight of miglitol. Further studies can be done by characterizing this degradation impurity to understand the possible impurities during the stability and the metabolites of miglitol.

### 3.3. Precision

All the individual values of impurity content and the assay in the precision and intermediate precision studies fell well in the range of confidence interval of average, confirming the excellent precision of the method. The recommended precision values in terms of percentage of relative standard deviation (RSD) should be not more than 15.0% for the related substance, whereas the percentage RSD of the content of impurities of miglitol in the method precision and the intermediate precision were within 5.2% and 2.28% respectively (Table 1).

### 3.4. Limit of Detection and Limit of Quantification

**Figure 13:**
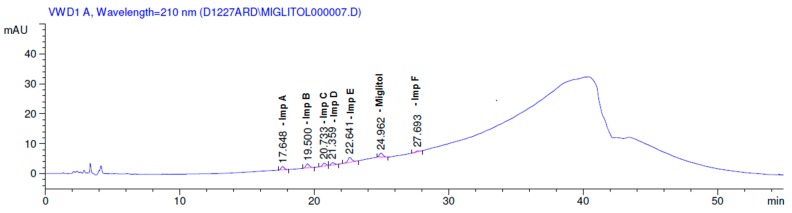
Limit of quantitation solution of miglitol and other impurities.

The limit of detection of miglitol was 0.02% (*w*/*w*) (of TAC) for 20 µL injection volumes. The limit of quantification for miglitol was 0.05% (*w*/*w*). Due to that, the response of all the impurities was quite good at 210 nm, the concentration at LOD and the LOQ of the impurities was very much encouraging (Figure 13). This revealed the capability of the method for the quantification of impurities at lower level not only during the analysis of the sample at quality control but also to control them during the optimization of the process.

### 3.5. Linearity

Excellent correlation was achieved for the regression line of miglitol and its related impurities from 0.05% (*w*/*w*) to 0.30% (*w*/*w)*. The correlation coefficient obtained for all the plots was greater than 0.997. The *Y*-intercept of each plot was below 1.5% of the response at 0.15% (*w*/*w*) level of each impurity. Linear calibration plot for the assay was obtained over the calibration ranges tested, i.e., 0.05% (*w*/*w*) to 0.30% (*w*/*w*). An excellent correlation exists between the peak area and concentration of miglitol by getting correlation coefficient greater than 0.999. Y-Intercept for the assay concentration is also supportive that the plot is going almost through the origin.

### 3.6. Accuracy

The recovery of each impurity fell in the range from 87.6% to 103.1% and recovery data at individual impurity at each level captured in Table 6. Individual assay value at each level in triplicate was close to the assay of miglitol reference standard evaluated. All the individual recovery value of the assay and impurities fell well within the confidence interval of mean values. Good recovery values reflected the exact values of RRF of impurities as well as the accuracy of the method.

### 3.7. Solution Stability and Mobile Phase Stability

The percentage RSD of area of all impurities during solution stability and mobile phase stability experiments were within 20%. No significant changes were experienced in the content of any of the impurity during solution stability and mobile phase stability experiments. The solution stability and mobile phase stability experiment data confirmed that the sample solutions and mobile phases used were stable up to 48 h. It is an advantage to have longer analysis time, which reduces the number of standard preparation in the quality control during regular analysis.

## 4. Conclusions

Chromatographic and sample preparation conditions were developed and validated for miglitol and its isomeric impurities. The method was validated for miglitol and its epimeric impurities with appropriate accuracy, precision, linearity, robustness, solution stability and mobile phase stability. Identification and characterization were done by using mass spectrometry techniques. This method can be used for the routine analysis of the drug and it can also be applied for the formulated product.

It is prudent to remain optimistic in the diabetes research, which is growing within the basic, translational and development segments. Novel technology may eventually help to improve the outcome and quality of the drugs. Many treatment options for diabetes like lifestyle modulation, diet control, obesity and medication are currently available to aid in the control and management of this disease.

Note: DRL-IPDO communication No.: IPDO IPM-00500 has been allotted for this research article in the research laboratory.

## Figures and Tables

**Table 1:** Limit of detection (LOD), limit of quantification (LOQ), regression and precision data.

Parameter	Miglitol	Imp A	Imp B	Imp C	Imp D	Imp E	Imp F
LOD % (*w*/*w*) w.r.t analyte concentration	0.02	0.02	0.02	0.02	0.02	0.02	0.02
LOQ % (*w*/*w*) w.r.t analyte concentration	0.05	0.05	0.05	0.05	0.05	0.05	0.05
Slope	385.7	540.08	365.7	305.3	676.3	612.5	164.9
Intercept	−0.271	0.833	0.875	0.121	0.124	−2.669	−1.22
Correlation coefficient	0.999	0.999	1.000	0.998	0.999	0.999	0.999
Method precision (% RSD)	5.21	3.54	1.76	3.44	1.49	5.28	6.91
Intermediate precision (% RSD)	2.28	0.87	0.54	2.62	4.90	3.56	3.43

W.r.t: with respect to RSD: relative standard deviation.

**Table 2:** Chemical name, structure and decoding list of miglitol and impurities.

Name of the Compound	Structure	Chemical Name	Emprical Formula
Miglitol	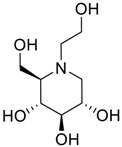	(2*R*,3*R*,4*R*,5*S*)-1-(2-hydroxyethyl)-2-(hydroxymethyl)piperidine-3,4,5-triol	C_8_H_17_NO_5_
Impurity A (Dialkylated Miglitol)	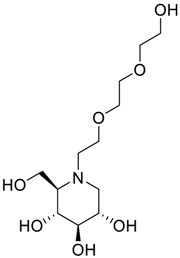	(2*R*,3*R*,4*R*,5*S*)-1-(2-(2-(2-hydroxyethoxy)ethoxy)ethyl)-2-(hydroxymethyl)piperidine-3,4,5-triol	C_12_H_25_NO_7_
Impurity B (Ido)	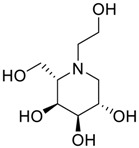	(2*S*,3*S*,4*R*,5*S*)-1-(2-hydroxyethyl)-2-(hydroxymethyl)piperidine-3,4,5-triol	C_8_H_17_NO_5_
Impurity C (Monoalkyl Miglitol)	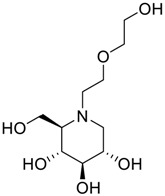	(2*R*,3*R*,4*R*,5*S*)-1-(2-(2-hydroxyethoxy)ethyl)-2-(hydroxymethyl)piperidine-3,4,5-triol	C_10_H_21_NO_6_
Impurity D (Taro)	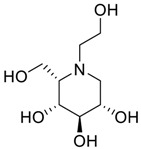	(2*S*,3*R*,4*R*,5*S*)-1-(2-hydroxyethyl)-2-(hydroxymethyl)piperidine-3,4,5-triol	C_8_H_17_NO_5_
Impurity E (Galacto)	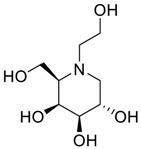	(2*R*,3*S*,4*R*,5*S*)-1-(2-hydroxyethyl)-2-(hydroxymethyl)piperidine-3,4,5-triol	C_8_H_17_NO_5_
Impurity F (Deoxynirijomycin)	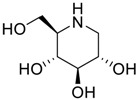	(2*R*,3*R*,4*R*,5*S*)-2-(hydroxymethyl)piperidine-3,4,5-triol	C_6_H_13_NO_4_
Miglitol *N*-oxide	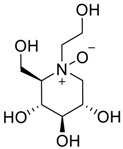	--	C_8_H_17_NO_6_

**Table 3:** Liquid chromatography mass spectrometry (LCMS) and tandem mass spectrometry (MS/MS) data of miglitol and other compounds.

	LCMS-MS/MS		
	[M + H]^+^	Collision energy (eV)	Fragmentation pattern (m/z)
Miglitol	208.2	20	190.10,172.10,154.10,146.10,74.10
		50	140.10,96.10,80.10,56.20
Imp-A	296.2	20	146.10,128.10,110.10,102.10,69.20,60.20
Imp-B	208.2	20	190.10,172.10,154.10,146.10,74.10
		50	140.10,96.10,80.10,56.20
Imp-C	252.2	20	234.10,190.10,172.10,118.10,86.10,58.20
Imp-D	208.2	20	190.10,172.10,154.10,146.10,74.10
		50	140.10,94.10,80.10,56.20
Imp-E	208.0	20	190.10,172.10,154.10,146.10,74.10
		50	140.10,94.10,80.10,56.20
Imp-F	164.2	20	146.10,128.10,110.10,69.20,60.20
MIG *N*-Oxide	224.2	20	176.10,146.10,74.20

**Table 4:** High resolution mass spectrometry (HRMS) data of miglitol (MIG) and other compounds.

Name of Compound	HRMS Data			
	[M + H]^+^	ppm	Double Bond Equivalance	Elemental Composition
Miglitol	208.1190	2.4	0.5	C_8_H_18_NO_5_
Imp-A	296.1696	−4.4	0.5	C_12_H_26_NO_7_
Imp-B	208.1185	−1.9	0.5	C_8_H_18_NO_5_
Imp-C	252.1435	−4.8	0.5	C_10_H_22_NO_6_
Imp-D	208.1177	−3.8	0.5	C_8_H_18_NO_5_
Imp-E	208.1189	1.9	0.5	C_8_H_18_NO_5_
Imp-F	164.0919	−2.4	0.5	C_6_H_14_NO_4_
MIG *N*-Oxide	224.1129	−2.2	0.5	C_8_H_18_NO_6_

**Table 5:** Summary of forced degradation results.

Stress condition	Duration	Purity of miglitol after forced degradation (%)	Content of major degradant (%)	Remarks
Acid hydrolysis	10 days	100	-	No degradation products formed
Base hydrolysis	10 days	100	-	No degradation products formed
Oxidation	1 h	0.0	100	Significant degradation product formed
Thermal (105 °C)	10 days	100	-	No degradation products formed
Photolytic as per ICH	11 days	100	-	No degradation products formed

**Table 6:** Results of accuracy for related substances.

Compound	Level	Concentration (% *w*/*w*)	Recovery in %	
			Individual	Mean
Imp-A	LOQ	0.05	99.2	95.65
	50%	0.075	96.3	
	100%	0.15	97.9	
	150%	0.225	89.2	
Imp-B	LOQ	0.05	100.2	96.03
	50%	0.075	97.6	
	100%	0.15	97.5	
	150%	0.225	88.8	
Imp-C	LOQ	0.05	101.3	99.95
	50%	0.075	101.7	
	100%	0.15	100.9	
	150%	0.225	95.9	
Imp-D	LOQ	0.05	93.0	97.58
	50%	0.075	99.1	
	100%	0.15	102.0	
	150%	0.225	96.2	
Imp-E	LOQ	0.05	94.0	96.63
	50%	0.075	94.5	
	100%	0.15	103.1	
	150%	0.225	94.9	
Imp-F	LOQ	0.05	91.0	95.25
	50%	0.075	99.3	
	100%	0.15	103.1	
	150%	0.225	87.6	
